# Community-Based Action Research Intervention to Promote Occupational Health Nursing of Portuguese Quarry Workers

**DOI:** 10.3390/nursrep14010030

**Published:** 2024-02-06

**Authors:** Catarina Magalhães Alves, Carminda Morais, Filipe Alves, Diogo Magalhães, Guilherme Gonçalves, Irma Brito

**Affiliations:** 1 Institute of Biomedical Sciences Abel Salazar (ICBAS), University of Porto, 4050 Porto, Portugal; 2 ISAVE—Superior Institute of Health, 4720 Braga, Portugal; 3 School of Health, Polytechnic Institute of Viana do Castelo, 4900 Viana Do Castelo, Portugal; carmindamorais@ess.ipvc.pt; 4 Nursing School of Coimbra & UICISA:E, 3045 Coimbra, Portugal; irmabrito@esenfc.pt; 5 Public Health Unit, Tâmega and Sousa Local Health Unit, 4630 Marco de Canaveses, Portugal; ftalves@arsnorte.min-saude.pt; 6 Matosinhos Local Health Unit, 4450 Matosinhos, Portugal; diogo.magalhaes@ulsm.min-saude.pt; 7 Local Health Unit of Médio Ave, 4765 Famalicão, Portugal; antonio.goncalves@arsnorte.min-saude.pt; 8 Executive Committee of International Collaboration for Participatory Health Research (ICPHR), 3045 Coimbra, Portugal

**Keywords:** occupational nursing, occupational health, health promotion, public health

## Abstract

The northern region of Portugal has the largest number of companies manufacturing granite and stone products, which has become the region’s trademark. In the municipalities of Marco de Canaveses and Penafiel, the economic activity of this area is important. However, the lack of attractiveness of this activity, combined with the high prevalence of silicosis and tuberculosis in this population, has led to a growing shortage of labor. In order for this project to be the result of collaborative, integral work centered on the people who are the target of health promotion, we used the Participatory Health Research (PHR) approach, based on the PRECEDE-PROCEED model, to implement a mixed-methods study, including participant observation, interviews and document analysis. These data were used to co-create a study design. In 2021, a total of 102 interviews were carried out and self-completion surveys were distributed: the Fantastic Lifestyle Questionnaire (FLQ) and the EQ-5D-3L. Within the scope of occupational health nursing and in the field of action of public health nurses, with the interviews and self-completed surveys carried out, we identified potential focuses for occupational health nursing intervention to promote the health of stone industry workers: adherence to protective measures, energy balance deficit, tobacco and alcohol consumption and access to health services. Data analysis made it possible to assess the prevalence of risk behaviors by order and to involve managers and workers in the co-creation of a health promotion program. The accurate identification of the focuses for nursing intervention not only improves the effectiveness of occupational health services, allowing for targeted interventions adapted to workers’ needs, but also contributes considerably to health promotion in the workplace, resulting in safer working environments, a reduction in occupational diseases and, consequently, a healthier and more productive workforce. This protocol of this study was not registered.

## 1. Introduction

In occupational health nursing in Portugal, nurses specialized in public health identify the needs of the target population with a view towards intervening to reduce morbidity and mortality and increase quality of life [1]. Promoting the health of stone industry workers is part of the National Occupational Health Program 2018–2020 [2], aiming at improving the quality of life, reducing early retirement due to disability and increasing the available human resources.

In the north of the country, many individuals work in the stone extraction and processing industry, especially of granite, an ornamental rock with a high silica content. The highest incidence of tuberculosis cases in the country is found in two of the municipalities where this industry is concentrated—Marco de Canaveses and Penafiel. In this setting, it is estimated that 30% of people with recently diagnosed tuberculosis (TB) work (or have worked) in stone quarries [3]. There is evidence of an increased risk of tuberculosis associated with housing conditions, precarious work, alcoholism and smoking, but also with pulmonary silicosis [4].

Considering the significant and progressive degradation of health of these stone processing workers, legislation was published in 2019 establishing a special regime for access to invalidity pensions and retirement at the age of 55 years [5].

In March 2018, in the municipalities of Marco de Canaveses and Penafiel, the project “Menos TB—pedreiras” (reducing TB—stone quarries) began, aimed at quarry workers. Periodic screenings of tuberculosis and latent tuberculosis infection were carried out in companies identified as being at higher risk of tuberculosis, with a view towards early diagnosis and treatment. A high prevalence of silicosis was also detected [4].

The promotion of workers’ health includes a process that is intended to enable people to improve control over their health [6]. This has traditionally been approached from a top-down perspective, in which health services identify risks and vulnerabilities and simultaneously propose strategies to improve health through protective measures and changes to the environment. Most occupational health studies focus on identifying specific problems that are assumed by health professionals to be known [7].

Nursing care should be coordinated in a collaborative, safe, high-quality, comprehensive and person-centered manner [1]. In the specific context of the stone industry, there is no scientific evidence of this investment in involving workers, because health promotion in this area has been almost non-existent or through a top-down logic. In 2020, in Portugal, 1366 trainings were carried out by the occupational health and safety services in the extractive industry and 77,457 trainings were carried out in the manufacturing industry. Of these, in the area of health promotion, only 16 were aimed at the extractive industry and 2176 were aimed at the manufacturing industry [8].

Health promotion must be based on four pillars: good governance (with the knowledge that health is influenced by many factors outside the control of the health sector, and that these can interfere with morbidity and mortality); health literacy (improving community health literacy will lead to more active community participation and improve individual health); healthy settings (which have community participation, empowerment, partnerships and equity as essential principles); and social mobilization (which will ensure both individual and community involvement) [6]. 

In the search for an approach that maximizes the participation of individuals throughout the process [7], focusing on their life and workstyles, as the object of this research and oriented towards change, Participatory Health Research (PHR) has been used, based on the PRECEDE-PROCEED model: “Predisposing, Reinforcing and Enabling Constructs in Educational Diagnosis and Evaluation- Policy, Regulatory, and Organizational Constructs in Educational and Environmental Development” [9]—a guide that facilitates and guides the planning and evaluation of health promotion programs. It translates into a cost–benefit evaluation framework, proposed in 1970 by Lawrence W. Green [10], and is applied worldwide to different population groups and for the development of health programs [11].

This model consists of eight phases [9]: social assessment, epidemiological assessment, educational/ecological assessment, administrative/policy assessment and intervention planning, implementation, process evaluation, impact evaluation and performance evaluation. The educational assessment of the PRECEDE-PROCEED model is classified into predisposing, reinforcing and facilitating factors that affect behavior and environmental changes [11]. The conclusion of the PRECEDE phase (health program design) leads to the beginning of the PROCEED phase, which deals with the implementation and evaluation of the health promotion program [9].

Using this model guarantees a collaborative diagnosis of the situation and activates health promotion processes with quarry workers, as part of cultural synthesis [6]. Needs, priorities and interventions are defined collaboratively, in a process of co-creation with and for the target people. The model also supports the action plan which contributes to meeting Sustainable Development Goals (SDGs) 2, 3 and 4: “end hunger, achieve food security and improved nutrition and promote sustainable agriculture”, “Ensure healthy lives and promote wellbeing for all at all ages” and “Ensure inclusive and equitable quality education and promote lifelong learning opportunities for all” [12] (p. 21).

This article describes the PRECEDE phase of identifying nursing intervention focuses, for the co-creation and implementation of a health promotion program for stone industry workers.

## 2. Materials and Methods

We implemented a PHR approach study [13] in three stone extraction and processing industry companies, with a total of 204 workers: company A—19 workers; company B—93; and company C—92. The companies were selected using a purposive sampling method and this study did not aim to compare companies. As this was a community-based action research project, no sample calculation rules or inclusion and exclusion criteria were applied, nor were the margins of error of the results from a statistical point of view. The data had participatory and contextual validity [13]. However, scientific and ethical rigor in data collection and analysis was respected and guaranteed. It should be noted that the research protocol was only submitted for approval by the Ethics Committee, after approval by the participating companies. 

The first phase (in 2019) involved visits to companies and meetings with employers and workers’ representatives, with the aim of presenting the project and gathering input for the co-creation of the study design. The main themes that emerged were the shortage of staff; the social perception of quarries as an unhealthy workplace; high worker turnover; high absenteeism; and the negative impact of exposure to dust since childhood, due to the fact that parents were stone quarry workers.

At the end of 2021, after a two-year interruption due to the COVID-19 pandemic, and applying the first stage of the PRECEDE-PROCEED model [10], mixed data collection techniques were used in the three stone quarries, including participant observation, document analysis, self-administered questionnaires and semi-structured interviews. We complied with the ethical requirements approved by the Ethics Committee (UICISA:E 796/06-2021) and obtained the free and informed consent of the 102 stone industry workers who took part. Data collection was carried out in the quarries by three specialists in community health nursing. The data collection protocol included sociodemographic characterization, a semi-structured interview on medical history and occupational risk, and the self-completion instruments “Fantastic Lifestyle Questionnaire (FLQ)”—validated for the Portuguese population [14] and the EQ-5D-3L [15]. The FLQ is a self-monitoring questionnaire made up of ten domains, with 30 closed-answer questions, where the person who completes it knows the result immediately. The sum of all the points (0, 1 or 2) results in an overall score between 0 and 120 points, which can be analyzed immediately, with the lowest scores enabling the immediate identification of intervention priorities, depending on the domain. The EQ-5D-3L is a generic instrument for assessing health-related quality of life and includes a visual analog scale, which makes it possible to determine the state of health at that precise moment (with “0” being the worst imaginable state of health and “100” the best imaginable state of health) [15].

The qualitative data were archived and recorded in the principal investigator’s field diary (meeting minutes, photos and audio recordings) and subjected to content analysis, i.e., building knowledge by analyzing the discourses, the layout and the terms used [16]. The quantitative data collected through the different instruments were analyzed in SPSS, using descriptive statistics. At various meetings, workers were invited to analyze the quantitative and qualitative results obtained, validating them (empathic validity, according to ICPHR, 2013).

## 3. Results

### 3.1. Phase 1: Social Assessment

A total of 102 people took part in this study, 50% of the total workforce of three companies: 19 from company A (100%), 44 from company B (47%) and 39 from company C (42%). Most participants were male (88%), with an average age of 38.8 ± SD 10.6 years, ranging from 18 to 66 years. The majority (63.4%) had an education up to the 9th grade. In addition, 75% lived in rural areas, with only 58.3% with drinking water from piped household connections to the public water supply and 63% with sanitation. Of the 102, 81.6% worked and lived in the same municipality. The average duration of employment in the stone industry was 15.6 ± SD 11.7 years, ranging from 1 to 46 years.

### 3.2. Phase 2: Epidemiological Assessment

Of the self-reported diseases classified according to the ICD-11 [17], 32 workers reported preconditions. Diseases of the circulatory system (39.0%) and respiratory system (22.2%) stood out, including silicosis (5.6%). Also above 10% were diseases of the digestive system (13.9%); the musculoskeletal system or connective tissue (13.9%); endocrine, nutritional or metabolic diseases (11.1%); and mental, behavioral or neurodevelopmental disorders (11.1%). Of the 32 workers who reported preconditions, 25% reported having more than one. The EQ-5D-3L showed that 42.0% reported chronic pain and 34.3% reported anxiety/depression. The average visual analog scale for describing health status was 79.7 ± SD 14.1 points, out of a total of 100 points. In the interview, 68% of the workers said that their parents worked or had worked in the stone industry and had been diagnosed with respiratory disease. Of those surveyed, 100% believed that there was a link between work activity and respiratory disease. The priority focuses for intervention were identified as monitoring chronic illness (32%), pain (42.0%) and mental health (34.3%).

### 3.3. Phase 3: Educational and Ecological Assessment

**Figure 1:**
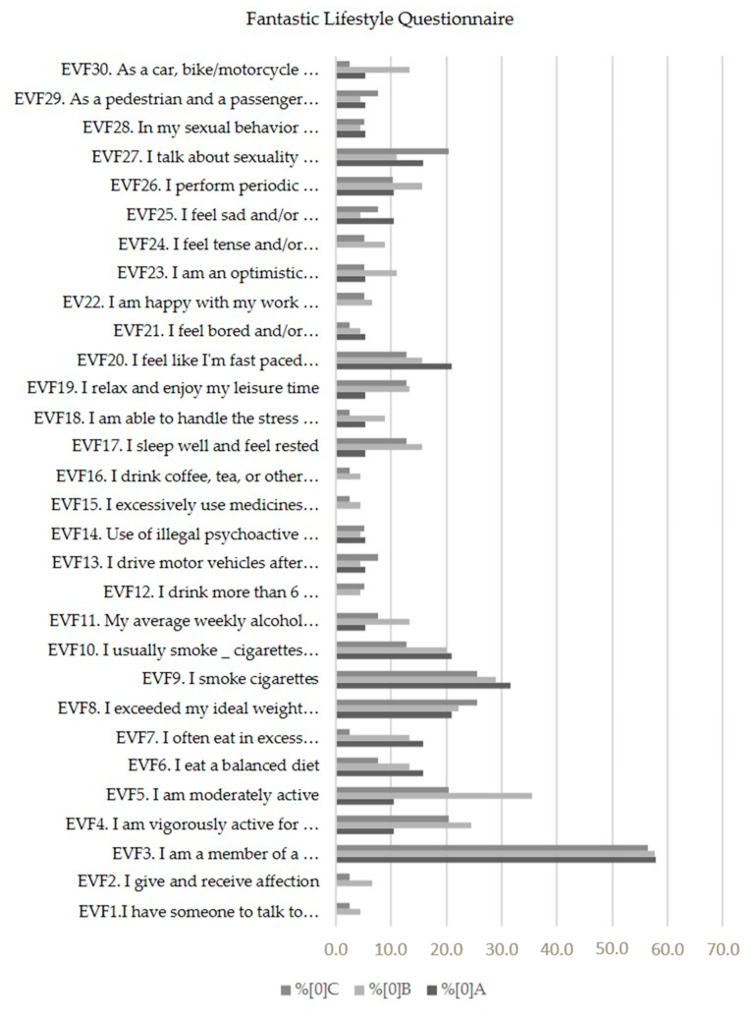
Results of the “Fantastic Lifestyle Questionnaire” (N = 102).

Regarding lifestyle, 45% of workers were overweight or obese [18] and 23.5% reported smoking, with consumption varying between 3 and 40 cigarettes a day, with 39.3% having tried quitting smoking at least once with no success. The average results of the FLQ by company were company A, 88.9; company B, 85.8; and company C, 97.2, which translates into a “Very Good” (85 to 102 points) or “Excellent” (103 to 120 points) lifestyle [14]. The domains most affected (Figure 1) were “physical activity/association”, “nutrition”, “tobacco” and “mental health”, with no significant differences between quarries. 

When workers were asked which personal protective equipment (PPE) was considered most important for their activity, there was a low perception of the importance of its use: mask (61.2%); boots/shoes (59.2%); ear protection (53.4%); and gloves (57.3%). Glasses/visors (23.3%), helmets (9.7%) and vests (2.9%) were not reported as important for workers’ individual protection. The visits and questionnaires identified various behavioral patterns requiring intervention. When asked about mask use in the pre- and post-pandemic period, use increased after the pandemic, from 60.3% to 82.4%.

When asked how they felt at the end of a day’s work, the answers were fatigue (78.4%), headaches (17.6%), feeling nervous (16.7%) and blocked nose/catarrh (15.7%). Observations of the environmental conditions in the quarries highlighted dust, noise from stone processing machines and trucks, and the exposure to adverse temperature and humidity conditions. The priority focuses for intervention were identified as follows: adherence to protective measures: use of PPE mask (38.8%), boots/shoes (40.8%), hearing protection (46.6%) and goggles/visor (76.7%), and polluted outdoor working environment and sometimes working in adverse weather.

The workers’ answers to the questions point to an understanding of health and safety recommendations as an asset for themselves (96%) and for the company. However, the lack of adherence to personal protective measures and the fact that only 10.7% of workers are not concerned about their health point to the social desirability of the answers. The lack of knowledge about the benefits of using PPE confirms the need for health promotion interventions.

As for their perception of excessive physical demand at work, 53.4% answered yes. The workers’ suggestions to reduce physical demand were as follows: use of machinery (14%), reduction in the size of the raw material (2%) and increasing human resources and reorganizing the workspace (1%). In addition, 35.9% of the workers who mentioned excessive physical demand could not identify any alternative to avoid it. The companies’ concern for the health of their workers was mentioned by 92% of respondents.

Regarding the frequency of the training provided by the health and safety occupational services, when asked if they attended training, 59.2% answered “always/often”, 9.7% answered “sometimes” and 31.1% answered “rarely/never”. A proportion of 51.5% said they attended training annually, 14.6% attended every 2 years, 11.7% had never attended, 8.7% attended only on admission, 2.9% attended every six months and 10.7% did not answer. Of the respondents who had attended training, 80% felt that it had met their needs, 17.5% said it had not met their needs and 2.5% did not answer. Of the 67 stone quarry workers, 89.3% said that the importance of wearing a mask had been explained to them when they were hired and 84.5% said that they knew how it should be worn correctly. When asked about the topics they would like to see covered in health and safety at work training, of the 40.8% of workers who responded, 28.2% mentioned first aid, 11.7% mentioned occupational safety techniques, 7.8% mentioned improving techniques and 4.9% mentioned training in machine handling.

When asked about their involvement in company decision making, 34% said they felt involved “sometimes” and 47.6% said “rarely/never”. It was found that 61.2% of the workers said that someone in their family had worked in the stone industry (76.2% were fathers or mothers and, of these, 67.7% said that during their childhood/youth, they stayed in the quarry with them). Of those that stayed regularly at the quarry during childhood, the average age at which they reported accompanying their parent was 11.6 ± SD 4.5 years, ranging from 5 years to 22 years. When asked about extra-occupational activities organized by the company, they mentioned Christmas dinner (33%), picnic day (5.8%) and the company’s anniversary (1.9%).

**Figure 2:**
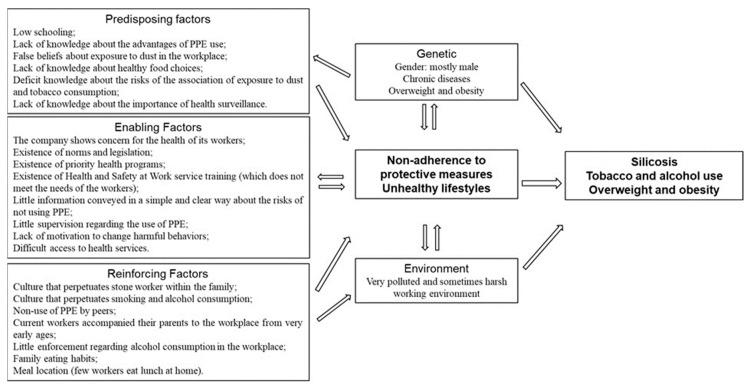
Predisposing, facilitating and reinforcing factors [9] (p. 151) of unhealthy lifestyles of stone industry workers.

Below is a schematic of the results of the PRECEDE phase, grouping them into predisposing, facilitating and reinforcing factors (Figure 2).

The priority focuses for intervention were a lack of knowledge about ergonomics (35.9%); a lack of access to health care, with 4.9% reporting never having had an occupational health consultation and 32.1% reporting that what they had was insufficient; and a lack of participation in decision making about health at work (47.6%). Culture also has an important influence on the perpetuation of non-adherence to protection.

### 3.4. Phase 4: Health Program and Policy Development

The visit to the premises revealed that there was a permissive attitude towards the non-use of personal protective measures and alcohol consumption at work. When workers were asked if they had made any suggestions to the company for improving health and safety at work, only 32% said they had. Some workers said they had suggested more than one measure, with 45.5% relating to working conditions, 18.2% relating to means and materials and 15.2% relating to means of protection (PPE). The implementation of the suggested measures was reported by 79% of workers.

When asked “If you were the boss, what would you change?”, 62% did not answer. Of the respondents, the changes suggested were to improve pay (11.7%), work conditions (9.7%), working materials (7.8%) and the organization of tasks (6.8%). When asked if they would like the company to set up a committee of worker’s representatives, 57% said yes and 5% did not answer. The fact that 62% did not answer the first question may reflect, on the one hand, the perception that each worker may have different expectations about change as bosses, which would lead to a variety of answers or the choice not to answer; on the other hand, it seems to demonstrate a sense of lack of individual power or influence to effect significant change, while the proposal of a committee may be perceived as a collective way of exerting influence, and may suggest a greater awareness of the importance of organized participation and collaborative work for improving working conditions. The priority focuses for intervention are the lack of zeal in complying with legislation.

The PRECEDE phase was concluded in meetings with workers where a list of priority focuses for occupational nursing intervention was finalized and discussed, common to the various companies, “Activity and Associations”, “Nutrition”, “Tobacco”, “Mental Health” and the non-use of personal protective equipment. In company “B”, “Alcohol and other substances”, “Introspection” and “Behavior on the road” also stood out.

## 4. Discussion

From the data collected from the 102 workers in the three stone industry companies, it is clear that they have a low level of schooling, which can influence their health literacy and consequently the ability to understand and adhere to health and safety measures at work. It is known that higher levels of education are associated with higher levels of health literacy [19] and that natural resource extraction activities have an associated cost in terms of environment and human health [20]. In this study, we found risk situations that are in line with SDG 4, the 2030 agenda for Portugal, which states that “trends have been less favorable for educational outcomes and competencies in some areas” [12] (p. 46), and which are the focus of occupational nursing interventions: a very polluted working environment and a lack of adherence to individual and collective protection measures.

A heavily polluted working environment is associated with the prevalence of respiratory system diseases. New technologies and new collective protective equipment might help reduce the amount of silica dust in suspension, but not to safe levels. As we show in this study, chronic health problems associated with respiratory diseases (22.2%), of which 5.6% are silicosis, are particularly worrying. Silicosis is probably the oldest recognizable occupational disease and has traditionally been related to mining or quarrying, but its incidence can be reduced by implementing strong worker protection measures and dust control [21]. In our study, we can see the need to improve the implementation of protective measures by focusing on behaviors associated with silicosis (low adherence to protection and safety measures at work) and cardiovascular disease (physical activity, nutrition, tobacco use, mental health), in order to contribute to reducing mortality from circulatory and respiratory diseases, within the scope of SDG 3 of the 2030 agenda for Portugal [12].

The analysis of predisposing, facilitating and reinforcing factors made it possible to identify the intensity of each factor, as well as to understand the complex interaction between them (Figure 2). Categorizing from this three-dimensional perspective makes it possible to organize nursing interventions based on the fact that people’s decisions, although rational, may not be the right ones for their needs. The self-assessment of their lifestyle as “Very Good” suggests that workers are aware of the importance of lifestyle to their health, but the implementation of healthy practices may need reinforcement.

The three priority problems identified through the FLQ are aligned with national data: physical activity, nutrition and tobacco. “Around a third (30%) of all deaths registered in Portugal in 2019 can be attributed to behavioral risk factors, namely smoking, dietary risks, alcohol consumption and low levels of physical exercise” [22] (p. 7). Portugal’s 2030 Agenda also mentions, in relation to SDG 2, “(…) unfavorable trends (…) with regard to diet” [12] (p.45). Evidence shows that behavioral factors such as low physical activity, unhealthy diet and smoking are major contributors to the burden of cardiovascular disease [23]. In 2019, 41% of Portuguese aged 16 and over suffered from at least one chronic disease, a higher proportion than in the EU (36%) [22].

The report “The Health of the Nation 1990–2016” states that risk factors are potentially modifiable causes of health loss. “The main risk factors for premature mortality in Portugal in 2016 are alcohol and drug consumption (…), inadequate diet, high blood pressure, tobacco consumption (…) pre-obesity and obesity.” [24] (p. 17), which corroborates our data. Our study identified substance use, smoking and alcohol consumption as problems in this population, with alcoholism being one of the most serious. Smoking cessation improves mental well-being by reducing anxiety, depression and stress [25], but employers from the companies in our study mentioned that they have no control over the use of tobacco and alcohol during working hours, nor do they have the ability to monitor the situation.

It should be noted that the lack of motivation to adhere to healthy lifestyles and to the use of PPE may be related to false beliefs/attitudes as well as a false sense of invulnerability. There is also little confidence in health and safety services, as workers report a moderate to poor availability of prevention and harm reduction services but a reasonable accessibility to general health services. The difficulty in involving employers in enforcing health and safety measures can be seen in the lack of compliance with legislation aimed at protecting workers’ health.

In our study, we identified a culture that perpetuates stone quarry working in the family, from an early age, which increases the risk of respiratory diseases. This is in line with the literature that shows that respiratory diseases are usually diagnosed in workers over 44 years of age—which may be associated with the need for a longer exposure time to develop the disease—and that many cases of silicosis are associated with a lack of adoption of preventive measures in silicogenic workplaces [26].

The stone quarry working context in Portugal is worrying and has probably not received sufficient attention, as illustrated by the fact that in 2020, there were only 16 health promotion actions at the national level for the extractive industry and 2176 for the manufacturing industry [27].

The average length of service in the stone industry (15.6 years) indicates an experienced working force, but at the same time suggests a greater cumulative exposure to occupational risks over many years. A low adherence to the use of personal protective equipment, especially masks, is a critical concern that needs to be addressed to protect the health of workers who carry out their activity in a potentially dangerous environment. The high percentage of workers who perceive health and safety at work as an asset to preserve their own health is encouraging. However, a sub-optimal understanding about the benefits of using PPE suggests the need to create specific health-promoting programs.

The low level of participation in decision making about health at work shows there is room for improvement in communication and worker involvement in health and safety policies and practices. Employers’ permissiveness for the non-use of PPE and alcohol consumption at work is a warning of the need to strengthen the application of safety policies.

## 5. Conclusions

This study had an innovative approach, involving the participants right from the definition of the health problem, and offers a comprehensive view of the working conditions and health of workers in the stone industry, in line with the responses to the sustainable development goals. A Participatory Health Research (PHR) approach was used, based on the PRECEDE-PROCEED model with mixed methods. Through participant observation, interviews and document analysis in three stone quarries, it was possible to identify focuses for occupational health nursing intervention (PRECEDE phase) in order to activate the co-creation and implementation of a program to promote the health of workers in the stone industry.

We found a population that is mostly poorly educated, affected by diseases of the circulatory and respiratory systems (in particular, silicosis—a disease associated with exposure to silica dust, which is particularly worrying and requires immediate intervention). The culture of perpetuating work in the stone industry within the family is an additional problem, as the time of exposure significantly increases—so this factor should also be considered when defining interventions. The failure to adopt preventive measures in silicogenic workplaces is an additional challenge that requires the active collaboration of companies to guarantee the health and safety of workers.

The results of this study also corroborate the concerns raised by employers, who seem to have been observing a decreasing interest in working in this kind of setting—as reflected in workforce shortages and high worker turnover.

Our study also provides a solid basis for involving workers in the co-creation and implementation of occupational health interventions, in order to improve health and increase the culture of safety in the stone industry, as well as define broader interventions related to behavioral risk factors, such as physical activity, nutrition, smoking and mental health.

## Data Availability

The data presented in this study are available on request from the corresponding author.

## Public Involvement Statement

No public involvement in any aspect of this research.

## Guidelines and Standards Statement

This manuscript was drafted against the Reporting of Primary Care Research: Consensus Reporting Items for Studies in Primary Care—the CRISP Statement for mixed-methods studies.
